# Unmasking the apoptotic potential of DHODH inhibition through targeting adaptive mitophagy

**DOI:** 10.3389/fcell.2026.1817489

**Published:** 2026-05-15

**Authors:** Xiaowen Huang, Zichang Guo, Bowen Liu, Hongyun Tang

**Affiliations:** 1 School of Life Sciences, Fudan University, Shanghai, China; 2 School of Life Sciences, Westlake University, Hangzhou, Zhejiang, China; 3 Westlake Laboratory of Life Sciences and Biomedicine, Hangzhou, Zhejiang, China

**Keywords:** apoptosis, ATG7, chloroquine, DHODH inhibition, mitophagy, mtROS

## Abstract

Targeting dihydroorotate dehydrogenase (DHODH) to restrict *de novo* pyrimidine synthesis is a promising anticancer strategy. However, the efficacy of DHODH inhibitors, such as brequinar (BQR), is often constrained by modest single-agent cytotoxicity, necessitating the exploration of combination therapies. Here, using mt-Keima-based mitophagy reporters and CRISPR/Cas9-mediated gene knockout models, we reveal a critical adaptive mechanism whereby BQR-induced mitochondrial reactive oxygen species (mtROS) trigger protective mitophagy. Crucially, we demonstrate that inhibiting this autophagy process synergistically enhances BQR’s anti-tumor activity both *in vitro* and *in vivo*. This combination leads to enhanced mtROS accumulation and severe lipid peroxidation, ultimately triggering caspase-dependent apoptosis, while ferroptosis does not appear to be the dominant mechanism under these conditions. Our findings identify mitophagy as a key mechanism of resistance to DHODH inhibition and provide a strong rationale for a combinatorial strategy to enhance the therapeutic efficacy of this class of drugs.

## 1 Introduction

Metabolic reprogramming is a hallmark of cancer, enabling tumor cells to meet the heightened biosynthetic and energetic demands associated with rapid proliferation and survival (Faubert et al., 2020). A key aspect of this adaptation is an accelerated rate of nucleotide biosynthesis. The *de novo* pyrimidine pathway is particularly critical, and its rate-limiting enzyme, dihydroorotate dehydrogenase (DHODH), offers a unique therapeutic vulnerability (Zhang et al., 2025; Anwar et al., 2025; Hai et al., 2024). As DHODH is localized to the inner mitochondrial membrane and functionally coupled to the electron transport chain, its inhibition not only blocks pyrimidine synthesis but also induces mitochondrial stress (Yang et al., 2023). Brequinar (BQR), a potent and selective DHODH inhibitor, has demonstrated antitumor activity in preclinical models across multiple cancer types. However, its efficacy as a monotherapy is often limited by insufficient cytotoxicity, highlighting the need for rational combination strategies to overcome resistance and enhance therapeutic outcomes (Madak et al., 2019; Peters et al., 1990; Zhang et al., 2022; Cao et al., 2025; Natale et al., 1992; Jiang et al., 2023).

Recently, an alternative, pyrimidine synthesis-independent role for DHODH has emerged, highlighting its function as a critical mitochondrial defense mechanism against lipid peroxidation and ferroptosis (Mao et al., 2021). Consequently, targeting DHODH to disrupt this defense axis has become a major therapeutic strategy for sensitizing cancer cells to ferroptosis (Hai et al., 2024; Mao et al., 2021; Yao et al., 2023; Cao et al., 2025; Peng et al., 2024). However, the efficacy of this approach is complicated by the fact that cellular defense against ferroptosis is primarily orchestrated by three parallel systems: Glutathione peroxidase 4 (GPX4), ferroptosis suppressor protein 1 (FSP1), and DHODH (Dixon and Olzmann, 2024). GPX4 and FSP1 function as parallel defense systems to inhibit lipid peroxidation, with GPX4 reducing toxic lipid hydroperoxides to alcohols while FSP1 primarily protects the plasma membrane (Dixon and Olzmann, 2024; Zhou et al., 2020). Mao et al. demonstrated that DHODH inhibition selectively induces ferroptosis only in cancer cells with low GPX4 expression, whereas GPX4-high cells remain resistant unless GPX4 is concurrently compromised (Mao et al., 2021; Mishima et al., 2023). Furthermore, Mishima et al. revealed a critical caveat: the pronounced ferroptosis-sensitizing effect of certain DHODH inhibitors at high doses is not mediated by DHODH blockade itself, but rather by the inadvertent, off-target suppression of FSP1(Mishima et al., 2023). Collectively, the anti-tumor efficacy of DHODH inhibitors via ferroptosis induction remains highly context-dependent. Beyond these known parallel systems, cancer cells may orchestrate other adaptive mechanisms to counteract DHODH-targeted stress.

Indeed, inhibition of DHODH is known to induce mitochondrial dysfunction, frequently resulting in the accumulation of mitochondrial reactive oxygen species (mtROS). To maintain organelle integrity and cellular homeostasis, cells activate quality control mechanisms, among which mitophagy, the selective autophagic degradation of damaged mitochondria, is paramount (Tábara et al., 2024). In cancer, mitophagy can serve as a pro-survival pathway, enabling tumor cells to alleviate metabolic and oxidative stress (Li et al., 2023). This raises an important question: does DHODH inhibitor-induced mitochondrial damage trigger a protective mitophagic response that attenuates its antitumor efficacy?

In this study, we demonstrate that BQR exposure elicits mtROS accumulation, which in turn activates a protective mitophagic flux. Crucially, we demonstrate that blocking this adaptive response, either through genetic ablation of *ATG7* or pharmacological lysosomal inhibition with chloroquine (CQ), synergizes with BQR by dramatically exacerbating mitochondrial oxidative stress and promoting potent, caspase-dependent apoptosis. Finally, we demonstrate that pharmacological inhibition of autophagy by CQ synergistically amplifies the anti-tumor activity of BQR in B16F10 melanoma mouse model *in vivo*. Collectively, our findings reveal that adaptive mitophagy constitutes a key resistance mechanism to DHODH inhibition and support the therapeutic potential of co-targeting DHODH and autophagy pathways to achieve superior anticancer effects.

## 2 Materials and methods

### 2.1 Cell lines and culture

The cell lines used in this study, including HEK293T (CRL-3216), HeLa (CCL-2), B16-F10 (CRL-6475), and 22Rv1 (CRL-2505), were obtained from the American Type Culture Collection (ATCC). HeLa, HEK293T, and B16-F10 cells were cultured in Dulbecco’s Modified Eagle Medium (DMEM; Gibco, 11965092) supplemented with 10% fetal bovine serum (FBS; Gibco, 10099141) and 1% penicillin-streptomycin (HyClone, SV30010). 22Rv1 cells were cultured in RPMI-1640 medium (Gibco, C11875500BT) supplemented with 10% FBS and 1% penicillin-streptomycin. Cells were maintained at 37 °C in a humidified incubator with 5% CO_2_. All cell lines were routinely tested for *mycoplasma* contamination and were confirmed to be negative.

### 2.2 Reagents and treatments

The following compounds were used at the indicated final concentrations: brequinar (BQR, 10 µM; MedChemExpress, HY-13751), chloroquine (CQ, 10 µM; Sigma-Aldrich, C6628), MitoTEMPO (10 µM; Sigma-Aldrich, SML0737), ferrostatin-1 (Fer-1, 10 µM; MedChemExpress, HY-100579), and Z-VAD-FMK (50 µM; MedChemExpress, HY-16658). In co-treatment experiments, cells were typically pretreated with the respective inhibitors (MitoTEMPO, CQ, Fer-1, or Z-VAD-FMK) for the indicated durations prior to BQR administration. DMSO was used as a vehicle control, with a final concentration not exceeding 0.1%.

### 2.3 CRISPR-Cas9 gene editing

Knocking out (KO) cell lines were generated using a two-plasmid CRISPR-Cas9 system. Tar-get-specific single guide RNA (sgRNAs) were designed using CRISPOR (crispor.tefor.net) and cloned into an sgRNA expression vector. sgRNA sequences are listed. HeLa cells were co-transfected with the sgRNA and Cas9 expression plasmids using Lipo8000™ transfection reagent (Beyotime). Following antibiotic selection, single-cell clones were isolated by limiting dilution in 96-well plates. Clones were expanded and screened by PCR amplification of the target genomic region followed by Sanger sequencing to identify frameshift mutations. Loss of target protein expression was confirmed by Western blotting. sgRNA sequences for gene knockout cell lines: *FIP200* sgRNA1: tat​gta​ttt​ctg​gtt​aac​ac; *FIP200* sgRNA2: aag​att​gct​att​caa​cac​c; *ATG7* sgRNA1: tat​ggg​aat​cca​taa​aat​cag​g; *ATG7* sgRNA2: gaa​ctt​gtt​gag​gag​tac​agg​g.

### 2.4 Western blotting

Cells were lysed in ice-cold RIPA buffer (Beyotime, P0013K) supplemented with protease and phosphatase inhibitor cocktails (Servicebio). Protein concentration was determined using a BCA assay kit (Beyotime, P0010). Equal amounts of protein (20 µg) were resolved by SDS-PAGE and transferred to PVDF membranes (Millipore, ISEQ00010). Membranes were blocked with 5% non-fat milk in TBST for 1 h at room temperature, followed by overnight incubation at 4 °C with the following primary antibodies: FIP200 (1:1000; CST, #12436S), ATG7 (1:1000; Abcam, #EPR6251), GPX4 (1:1000; Affinity, #DF6701), ACSL-4 (1:1000; GenuIN BIOTECH, #C61331), LC3B (1:1000, CST, #83506), Calnexin (1:2000, Proteintech #10427), Golgin-97 (1:2000, CST #13192T) and β-Actin (1:5000; Abclonal, #AC026). Membranes were incubated with HRP-conjugated secondary antibodies (Genscript) and visualized using a chemiluminescence imaging system (Bio-Rad).

### 2.5 Flow cytometry assays

All flow cytometry data were acquired on a CytoFLEX LX flow cytometer (Beckman Coulter) and analyzed using FlowJo software. A minimum of 10,000 events were recorded for each sample.

#### 2.5.1 Mitophagy assessment

HEK293T cells were seeded into 6-well plates at approximately 80% confluence and co-transfected with the transfer plasmid mt-Keima and lentiviral packaging plasmids (pRSV-Rev, Addgene#12253; pMDLg, Addgene#12251 and pCMV-VSV-G, Addgene#12259) using Lipo8000 transfection reagent (Beyotime, C0533). After 6–12 h, the medium was replaced with fresh complete medium. Viral supernatants were collected at 24–48 h after transfection, filtered through a 0.22-μm membrane, and supplemented with polybrene (10 μg/mL; Sigma-Aldrich, TR-1003). Target cells were infected by spinfection and subsequently subjected to fluorescence-activated cell sorting. Finally, we obtained stable HeLa-mtKeima, B16-F10 mt-Keima and 22Rv1 mt-Keima cell lines.

Mitophagic flux in cells stably expressing mt-Keima was quantified by measuring the ratiometric shift in Keima fluorescence. Cells were excited at 488 nm and 561 nm, and emission was collected using appropriate filters. An increase in the 561 nm/488 nm signal ratio indicates delivery of mt-Keima to the acidic lysosome (Sun et al., 2017; Wang, 2020).

#### 2.5.2 ER-phagy assessment

ER-phagy flux was quantified using HeLa cells stably expressing the mCherry-EGFP-RAMP4 tandem fluorescent reporter (Liang et al., 2018). During sample preparation, cell debris and doublets were excluded via forward scatter (FSC) and side scatter (SSC) gating. EGFP fluorescence was excited at 488 nm and collected using a 525/40 nm bandpass filter, whereas mCherry fluorescence was excited at 561 nm and detected with a 610/20 nm bandpass filter. Flow cytometry data were visualized on a dual-parameter scatter plot, with mCherry intensity on the Y-axis and EGFP intensity on the X-axis. Upon ER-phagy induction, the reporter is delivered to acidic lysosomes, where the EGFP signal is quenched (resulting in a significant decrease on the X-axis) while the mCherry signal remains stable. This causes a horizontal leftward shift of the cell population into the “Acidified ER” gating region. The proportion of Acidified ER-positive cells (calculated as the number of cells within the gate divided by the total number of analyzed cells) was measured to quantitatively evaluate the ER-phagy flux. Starvation induced by Earle’s Balanced Salt Solution (EBSS, Beyotome, C0213) served as a positive control.

#### 2.5.3 Mitochondrial ROS (mtROS) measurement

Cells were incubated with 5 µM MitoSOX™ Red (Thermo Fisher, M36008) in serum-free DMEM for 30 min at 37 °C. After incubation, cells were washed, harvested and immediately analyzed for red fluorescence (Miao et al., 2023).

#### 2.5.4 Lipid peroxidation assay

Lipid peroxidation was measured using the BODIPY™ 581/591 C11 sensor (Invitrogen, D3861). Cells were incubated with 5 µM BODIPY™ C11 for 30 min at 37 °C. Oxidation of the probe results in a shift from red to increased green fluorescence, which was quantified by flow cytometry.

#### 2.5.5 Apoptosis assay

Apoptosis was quantified using the FITC Annexin V Apoptosis Detection Kit I (BD Pharmingen, 556547) according to the manufacturer’s protocol. Briefly, cells were harvested, washed and resuspended in binding buffer before staining with Annexin V-FITC and Propidium Iodide (PI) for 15 min.

### 2.6 Confocal microscopy

Live-cell imaging was performed on a Nikon A1R confocal microscope. For mitophagy analysis, HeLa-mtKeima cells were imaged to detect pH-dependent shifts in Keima fluorescence. For colocalization studies, lysosomes were visualized by co-transfection with LAMP1-BFP.

### 2.7 Cell viability assay

Cell viability was measured using the Cell Counting Kit-8 (CCK-8; Beyotime). Cells were seeded in 96-well plates (3,000 cells/well), treated as indicated, and then incubated with CCK-8 reagent for 2 h at 37 °C. Absorbance was measured at 450 nm using a microplate reader (BMG Labtech).

### 2.8 Tumor xenograft animal experiments

Female C57BL/6 mice (8–10 weeks old) were obtained from Weitonglihua Laboratory Animal Co., Ltd. (Beijing, China) and maintained under specific pathogen-free conditions in a temperature-controlled facility with a regular 12-h light/dark cycle. All experimental procedures were approved by the Institutional Animal Care and Use Committee of Westlake University (protocol code: AP#22-071-11-THY-14). On day 0, mice were subcutaneously inoculated with 1 × 10^6^ B16-F10 cells suspended in 100 μL of PBS per animal and subsequently randomized into experimental groups. Mice received the following treatments on days 2, 6, and 10: double vehicle, Chloroquine monotherapy (CQ, 50 mg/kg, KKLMED, KM17589), Brequinar monotherapy (DMSO + BQR, 50 mg/kg, Amole, B209820), or the combinatorial treatment (CQ, 50 mg/kg + BQR, 50 mg/kg). All drugs and vehicles were administered via intraperitoneal injection. The experiment was terminated, and mice were euthanized on day 14.

### 2.9 Quantification of drug synergy

To quantitatively evaluate the pharmacological interaction between BQR and CQ, drug synergy was assessed using the Bliss independence model. First, the apoptosis rate (*D*) was normalized to determine the fractional effect (*E*) of each treatment (CQ alone, BQR alone, or in combination). This was calculated by adjusting for the background apoptosis rate of the vehicle control (*D*_*control*_) using the following equation: *E*_*treatment*_ = (*D*_*treatment*_ *- D*_*control*_)/(1 - *D*_*control*_). The net fractional effect (*E*) of each monotherapy was determined by subtracting the background apoptosis rate of the vehicle control from the treatment groups. The expected additive effect (*E*_*exp*_) of the combination treatment was calculated using the equation: *E*_*exp*_ = *E*_*CQ*_ + *E*_*BQR*_ -(*E*_*CQ*_ × *E*_*BQR*_). The Bliss Synergy Score (*S*) was then defined as the difference between the observed combination effect (*E*_*obs*_) and the expected additive effect, calculated as *S* = *E*_*obs*_ - *E*_*exp*_. A synergy score *S* > 0 indicates a synergistic interaction, *S* = 0 indicates an additive effect, and *S* < 0 indicates an antagonistic interaction (Foucquier and Guedj, 2015).

### 2.10 Statistical analysis

All quantitative data are presented as mean ± SEM from at least three independent biological replicates. Statistical analyses were performed using GraphPad Prism 9. Comparisons between multiple groups were conducted using one-way or two-way ANOVA with Tukey’s or Sidak’s post-hoc test for multiple comparisons. A p-value <0.05 was considered statistically significant. p-values are indicated in figures.

## 3 Results

### 3.1 BQR induces dose- and time-dependent mitophagy

**FIGURE 1:**
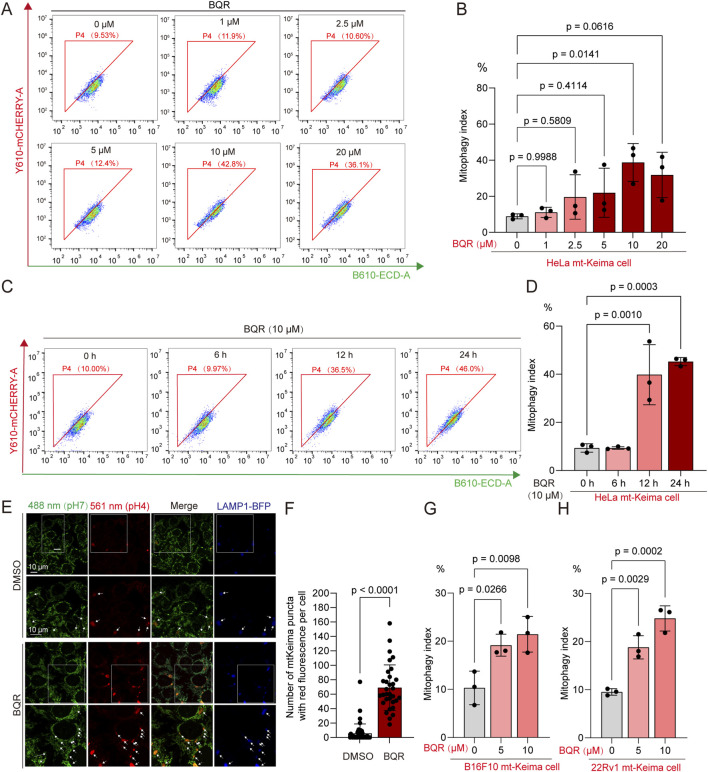
Brequinar (BQR) induces dose- and time-dependent mitophagy. **(A–D)** Mitophagy was quantified by flow cytometric analysis of the mt-Keima signal. **(A,B)** Dose-dependent mitophagy in HeLa mt-Keima cells treated with the indicated concentrations of BQR for 24 h **(C,D)** Time-dependent mitophagy in HeLa mt-Keima cells treated with 10 µM BQR for the indicated durations. *n* = 3 independent experiments. **(E,F)** Representative confocal images of HeLa mt-Keima cells treated with DMSO or 10 µM BQR for 24 h. Mitochondria (mt-Keima) are shown in green (neutral pH) and red (acidic pH). Lysosomes were labeled with LAMP1-BFP (blue) to confirm the delivery of mitochondria to lysosomes. **(E)** Representative confocal images. **(F)** Quantification of mt-Keima puncta (red fluorescence) per cell (*n* ≥ 30 cells per group from three independent experiments). Statistical significance was evaluated using a two-tailed Student’s t-test. **(G,H)** Mitophagy was quantified by flow cytometric analysis of the mt-Keima signal. **(G)** Dose-dependent mitophagy in B16-F10 mt-Keima cells treated with the indicated concentrations of BQR for 24 h. **(H)** Dose-dependent mitophagy in 22Rv1 mt-Keima cells treated with the indicated concentrations of BQR for 24 h *n* = 3 independent experiments. Data are presented as mean ± SEM from at least three independent experiments. Statistical significance was determined by one-way ANOVA unless otherwise specified. p-values are indicated.

Given that DHODH inhibition can disrupt mitochondrial redox homeostasis, we first sought to determine if the DHODH inhibitor BQR triggers a compensatory mitophagic response. To quantify mitophagic flux, we utilized HeLa cells stably expressing the mitochondria-targeted pH-sensitive reporter mt-Keima. Upon delivery to the acidic environment of the lysosome, mt-Keima undergoes a characteristic shift in its fluorescence spectrum (Katayama et al., 2011). Flow cytometric analysis demonstrated that BQR treatment led to a significant, dose- and time-dependent increase in the mitophagic index, with maximal induction of approximately 40% observed after 24 h of treatment with 10 μM BQR (Figures 1A–D; Supplementary Figure S1). Confocal microscopy confirmed these findings, revealing an increased accumulation of mitochondria within lysosomes, identified by the increased number of mt-Keima puncta with red fluorescence and their colocalization with the lysosomal marker LAMP1-BFP (Figures 1E,F). On the other hand, given that BQR specifically targets mitochondrial DHODH without altering the protein levels of Calnexin (ER marker) or Golgin97 (Golgi marker), or affecting ER-phagy flux (Supplementary Figure S1B–E), we demonstrated that BQR predominantly drives mitophagy. To investigate whether this is a universal phenomenon, we carried out parallel experiments using murine B16-F10 and human 22Rv1 cell lines. BQR induced increased mitophagy both in B16-F10 and 22Rv1 cells. Our consistent findings indicate that the induction of mitophagy by BQR is highly conserved across species and cell types (Figures 1G,H).

### 3.2 BQR-induced mitophagy is mediated by canonical macroautophagy

**FIGURE 2:**
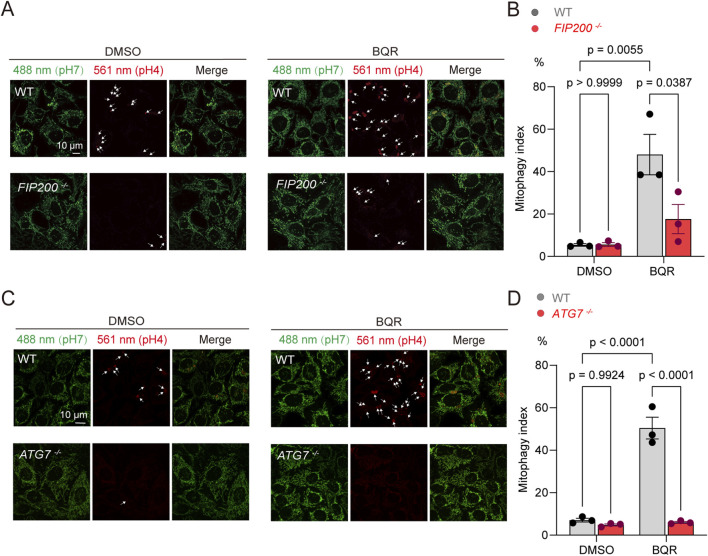
BQR-induced mitophagy requires the canonical macroautophagy components FIP200 and ATG7. **(A,B)** HeLa wild-type (WT) and *FIP200* ^−/−^ cells expressing mt-Keima were treated with 10 µM BQR for 24 h. **(A)** Representative confocal images. **(B)** Flow cytometric quantification of mitophagy. *n* = 3 independent experiments. **(C,D)** HeLa WT and *ATG7* ^*−/−*^ cells expressing mt-Keima were treated with 10 µM BQR for 24 h. **(C)** Representative confocal images. **(D)** Flow cytometric quantification of mitophagy. *n* = 3 independent experiments. Data are presented as mean ± SEM from at least three independent experiments. Statistical significance was determined by two-way ANOVA. p-values are indicated.

Next, we aimed to define the molecular machinery responsible for BQR-induced mitophagy. We investigated the role of the canonical macroautophagy pathway by generating knockout (KO) HeLa cell lines for FIP200 (*FIP200* ^*−/−*^), a core component of the autophagosome initiation complex, and ATG7 (*ATG7* ^*−/−*^), an E1-like enzyme essential for autophagosome elongation (Supplementary Figures S2A,B) (Liu et al., 2024; Nakatogawa, 2020). In stark contrast to wild-type (WT) cells, BQR treatment failed to induce mitophagy in both *FIP200* ^*−/−*^ and *ATG7* ^*−/−*^ cells, as measured by mt-Keima analysis via microscopy and flow cytometry (Figures 2A–D). These results demonstrate that BQR triggers mitophagy through the canonical, FIP200 and ATG7 dependent macroautophagy pathway.

### 3.3 BQR triggers mitophagy via mtROS accumulation

**FIGURE 3:**
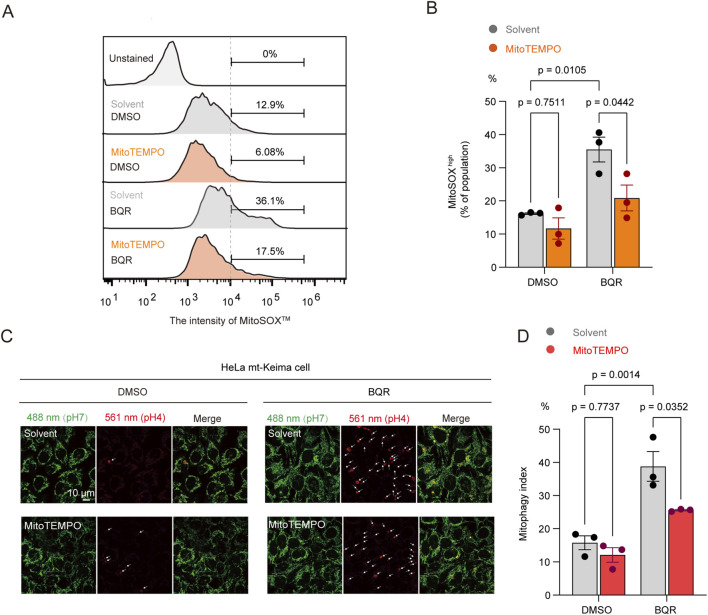
BQR-induced mitophagy is driven by mitochondrial ROS (mtROS). **(A,B)** HeLa cells were pretreated with vehicle or the mtROS scavenger MitoTEMPO (10 µM) for 12 h, followed by treatment with 10 µM BQR for 24 h mtROS was measured by MitoSOX™ Red staining. **(A)** Representative flow cytometry histograms. **(B)** Quantification of the proportion of MitoSOX^high^ cells. *n* = 3 independent experiments. **(C,D)** HeLa-mtKeima cells were treated as in **(A)**. **(C)** Representative confocal images. **(D)** Flow cytometric quantification of mitophagy. *n* = 3 independent experiments. Data are presented as mean ± SEM from at least three independent experiments. Statistical significance was determined by two-way ANOVA. p-values are indicated.

We then investigated the upstream signal initiating this mitophagic response. Because DHODH inhibition disrupts the mitochondrial electron transport chain, we hypothesized that the resulting mtROS act as the primary trigger (Schofield and Schafer, 2021; Cao et al., 2025). Consistent with this hypothesis, BQR treatment significantly increased mitochondrial superoxide levels measured by MitoSOX™ Red fluorescence. Specifically, BQR treatment significantly increased the proportion of MitoSOX^high^ cells, as evidenced by an approximately 1.7-fold increase compared to controls after 24 h (Figures 3A,B; Supplementary Figure S3). This effect was completely abrogated by pretreatment with the mitochondria-targeted antioxidant MitoTEMPO (Trnka et al., 2009) (Figures 3A,B). Crucially, MitoTEMPO pretreatment also abolished BQR-induced mitophagy (Figures 3C,D). These findings establish that mtROS accumulation is the necessary upstream signal for BQR-induced mitophagy.

### 3.4 Autophagy inhibition exacerbates BQR-induced oxidative stress and lipid peroxidation

Since mitophagy serves to clear damaged, ROS-producing mitochondria, we reasoned that inhibiting this protective process would amplify BQR-induced oxidative stress. To test this, we blocked general autophagic degradation either genetically (using *ATG7* ^*−/−*^ cells) or pharmacologically (with the lysosomal inhibitor chloroquine, CQ) (Mauthe et al., 2018; Cui et al., 2020). Autophagy inhibition by CQ was effectively confirmed by elevated LC3-II levels (Supplementary Figures S4A, B), as well as the ability of CQ to attenuate BQR-induced mitophagy (Supplementary Figure S4C).

**FIGURE 4:**
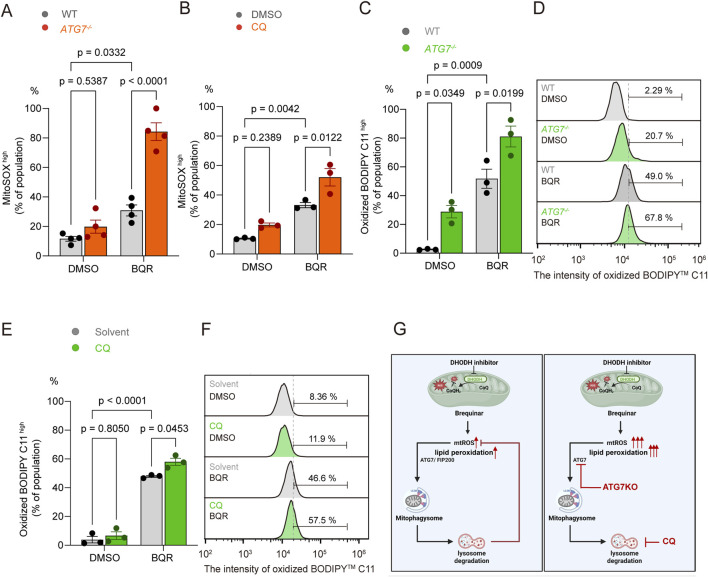
Autophagy Inhibition Exacerbates BQR-Induced Oxidative Stress and Lipid Peroxidation. **(A)** WT and *ATG7* ^*−/−*^ HeLa cells were treated with 10 µM BQR for 24 h and stained with MitoSOX™ Red. Quantification of the proportion of MitoSOX^high^ cells. *n* = 4 independent experiments. **(B)** WT HeLa cells were pretreated with the autophagy inhibitor chloroquine (CQ, 10 µM) for 12 h, followed by co-treatment with 10 µM BQR for 24 h. Quantification of the proportion of MitoSOX^high^ cells. *n* = 3 independent experiments. **(C–F)** Lipid peroxidation was measured using the BODIPY™ C11 sensor after 24 h of BQR (10 µM) treatment in **(C,D)** WT or *ATG7* ^*−/−*^ cells and in **(E,F)** WT cells treated with or without CQ (10 µM). Quantification of the proportion of Oxidized BODIPY™ C11 ^high^ cells. *n* = 3 independent experiments. **(G)** Schematic model illustrating the protective role of mitophagy in clearing ROS-producing mitochondria following DHODH inhibition by BQR. The schematic model was created with BioRender.com. Data are presented as mean ± SEM from at least three independent experiments. Statistical significance was determined by two-way ANOVA. p-values are indicated.

As hypothesized, both *ATG7* ablation and CQ co-treatment significantly augmented the accumulation of mtROS in BQR-treated cells compared to controls (Figures 4A,B). Specifically, under BQR treatment, genetic ablation of *ATG7* resulted in an approximately 2.7-fold increase in MitoSOX^high^ population compared to WT group. Likewise, pharmacological inhibition using CQ in combination with BQR induced a 1.7-fold increase in MitoSOX^high^ population relative to BQR treatment alone. Using the lipid peroxidation sensor BODIPY™ C11 (Drummen et al., 2002), we found that combining BQR with either *ATG7* ablation or CQ treatment led to a marked increase in lipid membrane oxidation (Figures 4C-F; Supplementary Figure S5) indicating severe oxidative damage. This demonstrates that mitophagy functions as a critical buffer against BQR-induced oxidative stress (Figure 4G).

### 3.5 Autophagy inhibition sensitizes cancer cells to BQR-induced apoptosis

**FIGURE 5:**
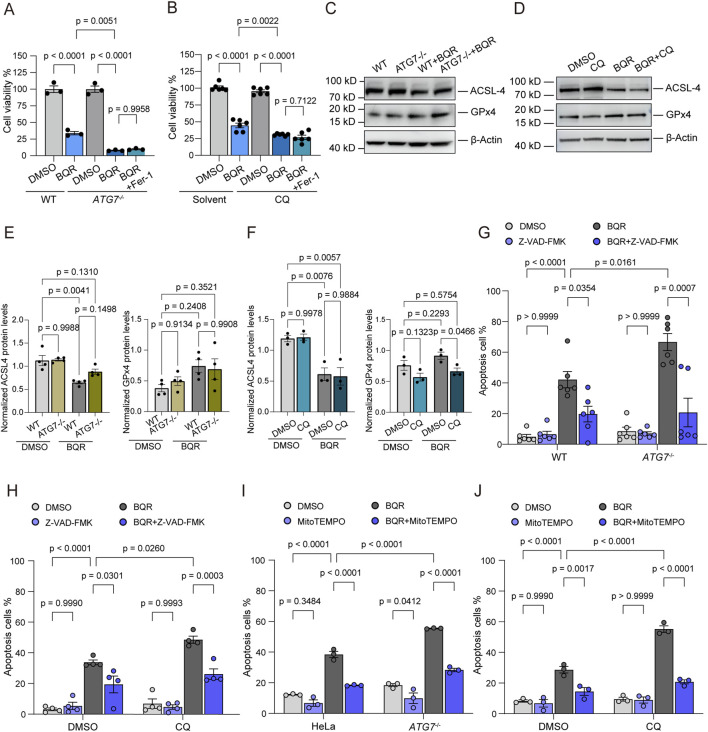
Autophagy Inhibition Sensitizes Cancer Cells to BQR-Induced Apoptosis **(A,B)** Cell viability was assessed by CCK-8 assay after 48 h of BQR (10 µM) treatment with or without the ferroptosis inhibitor ferrostatin-1 (Fer-1, 10 µM) in **(A)** WT or *ATG7* ^*−/−*^ cells, and in **(B)** WT cells co-treated with or without CQ. *n* = 3 independent experiments in A, *n* = 6 independent experiments in **(B)**. **(C)** Western blot analysis of ACSL-4 and GPX4 protein levels in wild-type (WT) and ATG7 knockout (*ATG7* ^*−/−*^) cells treated with DMSO (control) or BQR. β-Actin was used as a loading control. *n* = 3 independent experiments. **(D)** Western blot analysis of ACSL-4 and GPX4 protein levels in cells treated with vehicle (DMSO), the lysosomal inhibitor CQ alone, BQR alone, or the combination of BQR and CQ. *n* = 3 independent experiments. **(E)** Quantification of the Western blots shown in **(C)**, displaying the normalized protein expression levels of ACSL-4 and GPX4 relative to β-Actin. **(F)** Quantification of the Western blots shown in **(D)**, displaying the normalized protein expression levels of ACSL-4 and GPX4 relative to β-Actin. **(G)** WT and *ATG7* ^*−/−*^ HeLa cells were treated with or without 10 µM BQR for 48 h, in the presence or absence of the pan-caspase inhibitor Z-VAD-FMK (50 µM). Quantification of apoptotic cells (Annexin V^+^). *n* = 6 independent experiments. **(H)** WT HeLa cells were treated with BQR and Z-VAD-FMK as in **(G)**, with the addition of CQ (10 µM). Quantification of apoptotic cells (Annexin V^+^). *n* = 4 independent experiments. **(I)** WT and *ATG7* ^*−/−*^ HeLa cells were pretreated with MitoTEMPO (10 μM) for 12 h, followed by treatment with or without 10 µM BQR for 48 h. Quantification of apoptotic cells (Annexin V^+^). *n* = 3 independent experiments. **(J)** WT HeLa cells were pretreated with MitoTEMPO (10 μM) for 12 h, followed by treatment with or without 10 µM BQR for 48 h in the presence of CQ (10 µM). Quantification of apoptotic cells (Annexin V^+^). *n* = 3 independent experiments. Data are presented as mean ± SEM from at least three independent experiments. Statistical significance was determined by two-way ANOVA. p-values are indicated.

This enhanced oxidative damage correlated with a dramatic reduction in cell viability. Both *ATG7* ^*−/−*^ and CQ-treated cells exhibited significantly greater sensitivity to BQR-induced cytotoxicity after 48 h (Figures 5A,B; Supplementary Figures S6A, B). Despite the pronounced increase in lipid peroxidation, the specific ferroptosis inhibitor ferrostatin-1 (Fer-1) failed to rescue this cell death (Figures 5A,B) (Mao et al., 2021; Mishima et al., 2023; Jiang et al., 2023). To further validate this at the molecular level, we examined two core protein markers of ferroptosis: ACSL-4 (a crucial pro-ferroptotic enzyme) and GPX4 (a key anti-ferroptotic protector). Western blot analysis revealed no typical ferroptotic changes. Instead, BQR treatment significantly downregulated ACSL-4 expression, while GPX4 levels remained largely unaffected by autophagy inhibition (Figures 5C–F). Taken together, these results suggest that ferroptosis does not appear to be the dominant mechanism under these conditions.

Given that severe oxidative stress can also trigger apoptosis (Chen et al., 2025), we next assessed for markers of programmed cell death. Annexin V/PI staining revealed a substantial increase in the apoptotic population when mitophagy was blocked in BQR-treated cells (Figures 5G,H; Supplementary Figures S7A–C). Specifically, under BQR treatment, genetic ablation of *ATG7* resulted in an approximately 1.5-fold increase in the apoptotic population compared to WT cells (Figure 5G; Supplementary Figure S7B). Likewise, pharmacological inhibition using CQ in combination with BQR produced similar effects (Figure 5H; Supplementary Figure S7C). Furthermore, Bliss independence analysis confirmed that the combination of BQR and CQ exerts a significant synergistic lethality, yielding an average Synergy Score of 0.125 (12.5%). Critically, this synergistic lethality was almost completely rescued by the pan-caspase inhibitor Z-VAD-FMK (Figures 5G,H; Supplementary Figures S7B, C). On the other hand, under BQR treatment, the exacerbated apoptosis induced by the ablation of *ATG7* could be reversed under the treatment of mtROS scavenger MitoTEMPO (Figure 5I). Similarly, the exacerbated apoptosis induced by CQ in combination with BQR could also be reversed by MitoTEMPO treatment (Figure 5J). Taken together, these results demonstrate that inhibiting the protective mitophagic response converts BQR into a potent inducer of apoptosis by causing an overwhelming accumulation of mitochondrial oxidative stress.

### 3.6 Pharmacological blockade of autophagic degradation by CQ amplifies BQR induced apoptosis across diverse cancer models

**FIGURE 6:**
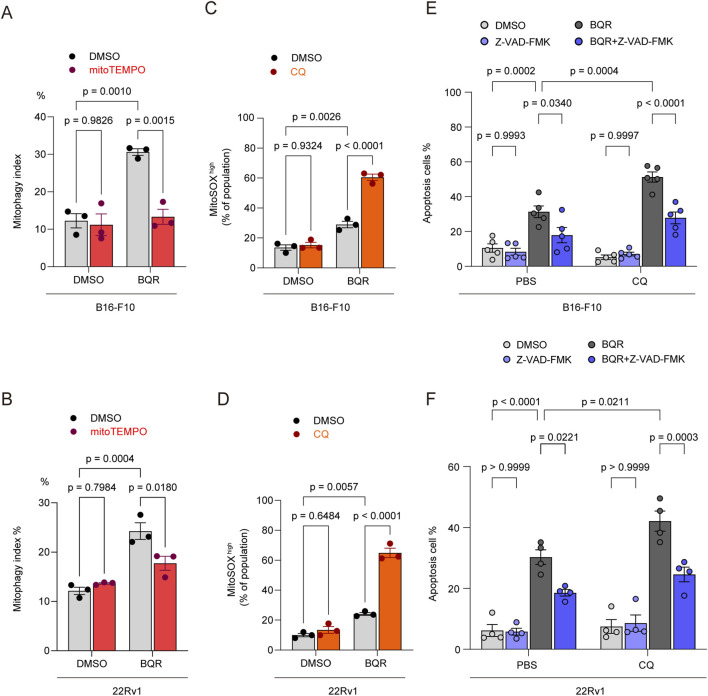
Pharmacological blockade of autophagic degradation by CQ amplifies BQR induced apoptosis across diverse cancer models. **(A)** Flow cytometric quantification of mitophagy. B16-F10 mt-Keima cells were pretreated with vehicle or 10 μM MitoTEMPO for 12 h, followed by treatment with 10 μM BQR for 24 h or not. *n* = 3 independent experiments. **(B)** Flow cytometric quantification of mitophagy. 22Rv1 mt-Keima cells were pretreated with vehicle or 10 μM MitoTEMPO for 12 h, followed by treatment with 10 μM BQR for 24 h or not. *n* = 3 independent experiments. **(C)** WT B16F10 cells were pretreated with10 μM CQ for 12 h or not, followed by co-treatment with 10 μM BQR for 24 h or not. mtROS was measured by MitoSOX_TM_ Red staining. Quantification of the proportion of MitoSOX high cells. *n* = 3 independent experiments. **(D)** WT 22Rv1 cells were pretreated with 10 μM CQ for 12 h or not, followed by co-treatment with 10 μM BQR for 24 h or not. mtROS was measured by MitoSOX_TM_ Red staining. Quantification of the proportion of MitoSOX high cells. *n* = 3 independent experiments. **(E)** WT B16-F10 cells were pretreated with 10 μM CQ for 12 h or not, followed by co-treatment with 10 μM BQR for 48 h in the presence or absence of 50 μM Z-VAD-FMK. Quantification of apoptotic cells (Annexin V_+_). *n* = 5 independent experiments. **(F)** WT 22Rv1 cells were pretreated with 10 μM CQ for 12 h or not, followed by co-treatment with 10 μM BQR for 48 h in the presence or absence of 50 μM Z-VAD-FMK. Quantification of apoptotic cells (Annexin V_+_). *n* = 4 independent experiments. Data are presented as mean ± SEM from at least three independent experiments. Statistical significance was determined by two-way ANOVA. p-values are indicated.

To determine whether our proposed mechanistic model is a generalizable phenomenon, we extended our investigation to the murine melanoma cell line B16-F10 and the human prostate cancer cell line 22Rv1. Consistent with our initial observations, BQR treatment significantly upregulated the mitophagy index in both cell lines. This protective mitophagic response was highly dependent on mitochondrial oxidative stress, as it could be largely abrogated by the mtROS scavenger MitoTEMPO (Figures 6A,B). Furthermore, when this autophagic clearance was pharmacologically disrupted using CQ, we observed a dramatic exacerbation of mtROS accumulation (indicated by the MitoSOX high population) in BQR-treated cells (Figures 6C,D).

Consequently, this unresolvable oxidative stress translated into profound cytotoxicity. The dual treatment of BQR and CQ triggered massive apoptosis in both B16-F10 and 22Rv1 cells, significantly surpassing the effect of BQR alone. Bliss independence analysis confirmed that the combination of BQR and CQ exerts a significant synergistic lethality, yielding an average Synergy Score of 0.271 (27.1%) in B16-F10 cells and an average Synergy Score of 0.115 (11.5%) in 22Rv1 cell. Crucially, this synergistic lethality was effectively reversed by the co-administration of the pan-caspase inhibitor Z-VAD-FMK (Figures 6E,F). Collectively, these results highlight a conserved therapeutic strategy: blocking autophagy sensitizes diverse cancer models to BQR-induced apoptosis.

### 3.7 Pharmacological blockade of autophagic degradation by CQ amplifies the anti-tumor efficacy of BQR *in vivo*

**FIGURE 7:**
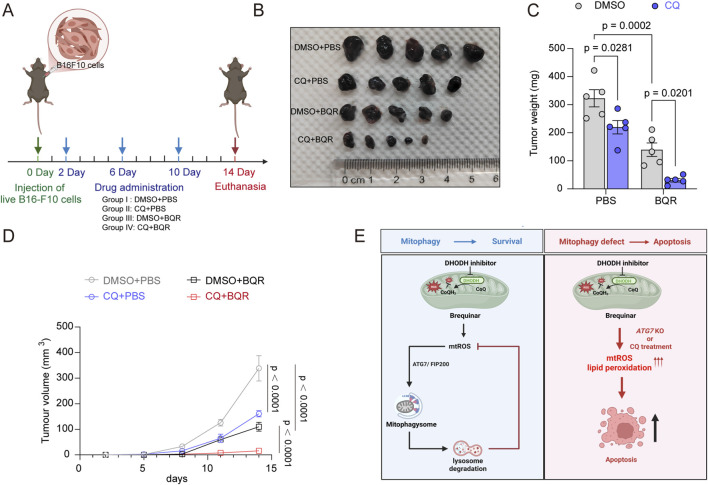
Pharmacological blockade of autophagic degradation by CQ amplifies the anti-tumor efficacy of BQR *in vivo* **(A)** Experiment schematic to test anti-tumor efficacy of combining BQR and CQ in subcutaneous B16F10 melanoma mouse model. On day 0, mice were subcutaneously injected with live B16F10 cells. Mice were randomized into four groups and received the following treatments on days 2, 6, and 10: double vehicle, CQ monotherapy (CQ 50 mg/kg), BQR monotherapy (BQR 50 mg/kg), or the combinatorial treatment (CQ 50 mg/kg + BQR 50 mg/kg). All drugs and vehicles were administered via intraperitoneal injection. The experiment was terminated, and mice were euthanized on day 14. **(B)** Representative images of excised subcutaneous melanoma tumors in four groups. (*n* = 5 mice per group) **(C)** Tumor weights in the different groups as indicated in **(A)**. **(D)** Tumor volumes over time for the groups indicated in **(A)**. **(E)** Schematic illustration of unmasking the apoptotic potential of DHODH inhibition through targeting adaptive mitophagy. DHODH inhibition by BQR triggers a compensatory mtROS surge, which recruits protective mitophagy to buffer mitochondrial oxidative stress and maintain cell survival. Genetic or pharmacological disruption of this mitophagic safeguard precipitates a catastrophic accumulation of mtROS and lipid peroxidation, effectively converting a manageable redox shift into a terminal apoptotic crisis. Created with BioRender.com. Data are presented as mean ± SEM from at least three independent experiments otherwise specified. Statistical significance was determined by two-way ANOVA. p-values are indicated.

To determine whether the synergistic cytotoxicity observed *in vitro* could be translated into anti-tumor efficacy *in vivo,* we evaluated the therapeutic efficacy of combining BQR and CQ in a subcutaneous B16F10 melanoma mouse model. Mice were randomized into four groups and received the following treatments: double vehicle, CQ monotherapy, BQR monotherapy, or the combinatorial treatment (CQ + BQR) (Figure 7A). The results showed that while BQR monotherapy reduced tumor weights by approximately 50% compared to the vehicle control, the addition of CQ further diminished tumor burden by nearly 90% (Figures 7B,C). Bliss independence analysis confirmed that the combination of BQR and CQ exerts a significant synergistic anti-tumor effect, yielding an average Synergy Score of 0.210 (21.0%) *in vivo*. Correspondingly, the results of tumor volumes over time demonstrated that the combination of CQ and BQR synergistically and durably suppressed melanoma progression (Figure 7D). Taken together, these *in vivo* findings support the therapeutic relevance of the mechanism observed *in vitro*, highlighting a highly potent combinatorial strategy for cancer therapy.

## 4 Discussion

Inhibiting DHODH represents a promising class of anticancer strategy, yet the clinical potential has been hampered by their limited cytotoxicity (Rajamohamed and Veerappapillai, 2025; Jiang et al., 2023). Our study provides a key mechanistic explanation for this limitation, revealing that cancer cells activate a protective mitophagic response to counteract the mitochondrial stress induced by the DHODH inhibitor BQR. We demonstrate that this process is driven by mtROS accumulation and dependent on the core autophagy protein ATG7. Crucially, by disabling this survival pathway, we unmask the latent cytotoxic potential of BQR (Figure 7E).

A salient and intriguing finding of our study is that while this synergistic combination induces lipid peroxidation, a canonical hallmark of ferroptosis, the ultimate cell fate is apoptosis. This suggests a potential hierarchy or threshold effect in the cellular response to oxidative damage. It is plausible that while the accumulated lipid peroxides are significant, they may not reach the specific threshold required to execute ferroptotic cell death. Alternatively, the overwhelming mitochondrial insult caused by the dual blockade may preferentially and more rapidly engage the intrinsic apoptotic cascade through the release of pro-apoptotic factors like cytochrome c, preempting the execution of ferroptosis (Glover et al., 2024). This distinction is critical, as it clarifies that the therapeutic synergy is driven by caspase activation and offers a clear mechanistic endpoint for future translational studies.

Our findings have direct translational implications. By identifying adaptive mitophagy as a key resistance mechanism, we establish a strong rationale for combining DHODH inhibitors with autophagy inhibitors to improve therapeutic outcomes. The co-administration of DHODH inhibitors with clinically approved lysosomal inhibitors like CQ emerges as a readily translatable strategy. This work provides the preclinical mechanistic basis for such a combination, suggesting it could overcome the inherent resistance observed with DHODH monotherapy and maximize tumor cell killing.

There are several limitations in our models. Although we demonstrated that chloroquine (CQ) treatment or ATG7 ablation exacerbates mtROS accumulation and subsequent cell death, these interventions broadly inhibit general autophagy. Therefore, we can only conclude that general autophagy blockade sensitizes tumor cells to BQR, but we cannot definitively attribute this therapeutic vulnerability exclusively to mitophagy inhibition. A key priority is to identify the specific mitophagy receptors that are essential for recognizing BQR-damaged mitochondria. A more precise, receptor-level blockade could offer a more targeted and potentially less toxic means of disrupting this adaptive pathway compared to general autophagy inhibitors (Yang et al., 2024). Second, although Fer-1 failed to rescue cell viability and key ferroptotic marker proteins remained unchanged, we cannot completely rule out the partial involvement of ferroptosis in the cell death induced by the combination of BQR and autophagy inhibition. Further investigations, such as assessing iron dependency, are required to fully elucidate the exact role of ferroptosis in our model. Moreover, despite the robust anti-tumor effects of BQR and CQ cotreatment observed *in vivo*, we acknowledge the lack of direct mechanistic validation (such as apoptosis markers, mitophagy markers or mtROS levels) within tumor tissues to fully bridge the *in vitro* findings with the *in vivo* outcomes. Finally, exploring whether this mitophagic response is a general feature of other DHODH inhibitors would be valuable.

In conclusion, this study decodes a critical adaptive pathway that enables cells to survive DHODH inhibition. By demonstrating that blockade of mitophagy synergistically unleashes the apoptotic potential of BQR through overwhelming mtROS accumulation, we provide a robust mechanistic framework for developing superior combination therapies in oncology.

## Data Availability

The raw data supporting the conclusions of this article will be made available by the authors, without undue reservation.
