# Supplementary material for: Unmasking the apoptotic potential of DHODH inhibition through targeting adaptive mitophagy
Source: Front Cell Dev Biol. 2026 May 15;14:1817489. doi: 10.3389/fcell.2026.1817489 (PMC13220695; doi:10.3389/fcell.2026.1817489)
Supplement: Supplementary file 1 [file DataSheet1.pdf]

Supplementary Material

Unmasking the apoptotic potential of DHODH inhibition through targeting adaptive mitophagy

Xiaowen Huang[1,2], Zichang Guo[2], Bowen Liu[2,3*], Hongyun Tang [1,2,3*]

Figure S1

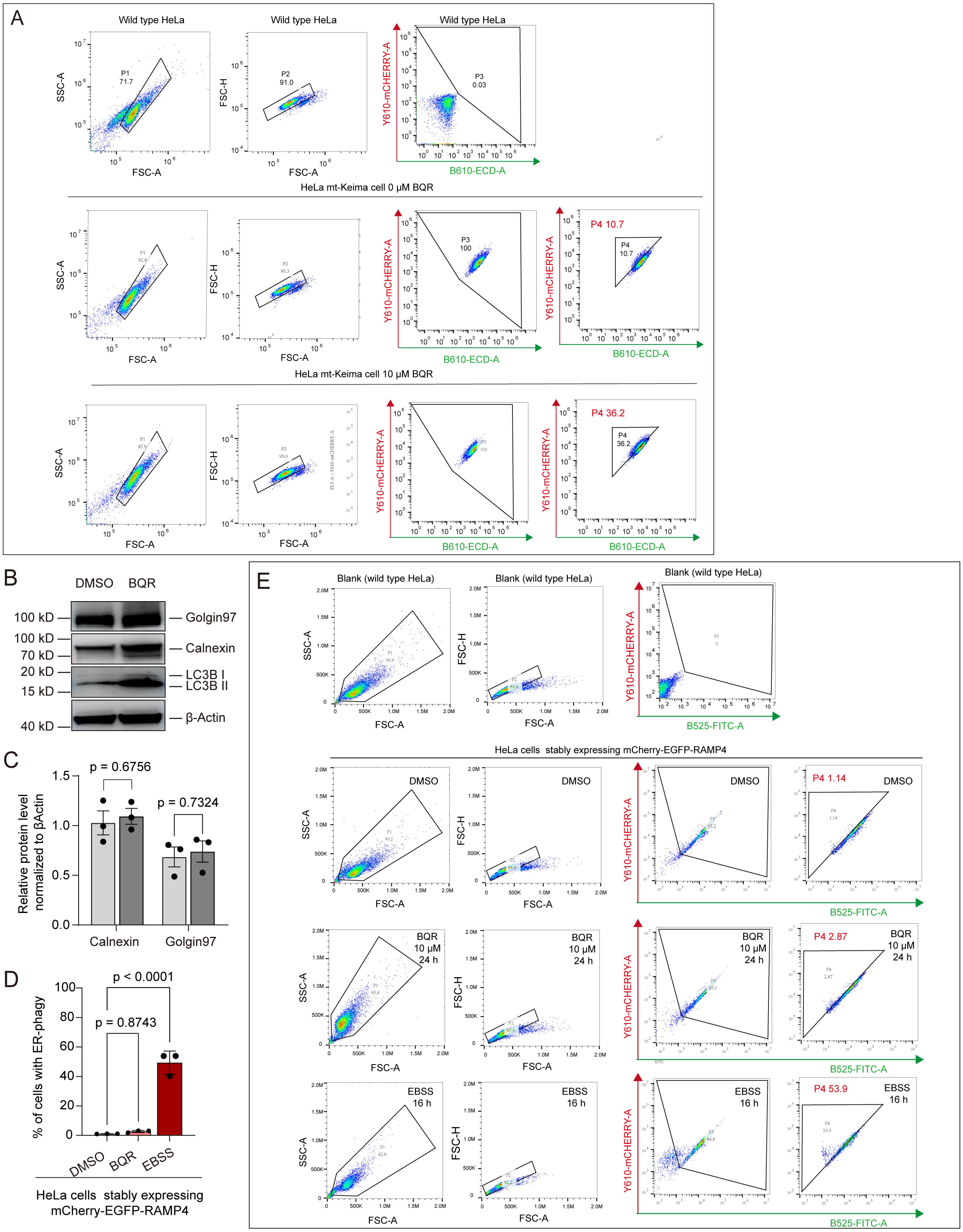
A
Wild type HeLa
HeLa mt-Keima cell 0 μM BQR
HeLa mt-Keima cell 10 μM BQR
SSC-A
FSC-A
FSC-H
Y610-mCHERRY-A
B610-ECD-A
P1 71.7
P2 91.0
P3 0.03
P3 100
P4 10.7
P4 36.2
B
DMSO
BQR
100 kD
70 kD
20 kD
15 kD
40 kD
Golgin97
Calnexin
LC3B I
LC3B II
β-Actin
C
Relative protein level normalized to βActin
p = 0.6756
p = 0.7324
Calnexin
Golgin97
D
% of cells with ER-phagy
p < 0.0001
p = 0.8743
DMSO
BQR
EBSS
HeLa cells stably expressing mCherry-EGFP-RAMP4
E
Blank (wild type HeLa)
HeLa cells stably expressing mCherry-EGFP-RAMP4
B525-FITC-A
P4 1.14
P4 2.87
P4 53.9
BQR 10 μM 24 h
EBSS 16 h

Figure S1 BQR induces mitophagy predominantly

(A) Gating strategy for mtKeima-based mitophagy analysis by flow cytometry. Representative flow cytometric plots illustrating the hierarchical gating and analytical logic for measuring mitophagy flux. Cell populations were first identified by FSC-A versus SSC-A to exclude debris. Single cells were isolated using FSC-A versus FSC-H. Mitophagy flux was quantified by calculating the ratio of lysosomal (561 nm excitation) to cytosolic (488 nm excitation) mt-Keima signals. Representative data are shown for HeLa cells treated with DMSO or 10 μM BQR for 24 hours.

(B) Representative Western blot of the protein expression levels of the Golgi marker (Golgin), endoplasmic reticulum marker (Calnexin), autophagy marker (LC3B), and the loading control (β-Actin) in the DMSO control and BQR-treated groups.

(C) Quantitative analysis of the relative protein expression levels of Calnexin and Golgin (normalized to β-Actin) in (B). $n = 3$ independent experiments. Statistical significance was evaluated using a two-tailed Student's t-test.

(D) Quantitative analysis of ER-phagy by flow cytometry. ER-phagy flux was evaluated in mCherry-EGFP-RAMP4-expressing HeLa cells treated with DMSO, BQR (10 μM, 24 hours), or EBSS (16 hours). The y-axis indicates the percentage of "Acidified ER" cells out of the total analyzed population, characterized by decreased EGFP and stable mCherry fluorescence upon lysosomal delivery. Starvation induced by EBSS served as a positive control. $n = 3$ independent experiments

(E) Schematic of the flow cytometric gating and analytical logic for ER-phagy flux measurement.

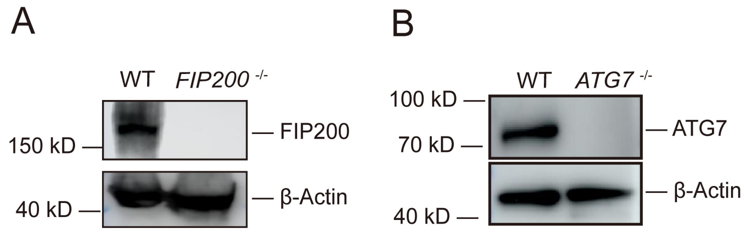

Figure S2 Validation of FIP200 and ATG7 deficiency.

(A, B) Western blot confirming the absence of FIP200 (A) and ATG7 (B) in HeLa cells. β-Actin served as a loading control. $n = 3$ independent experiments.

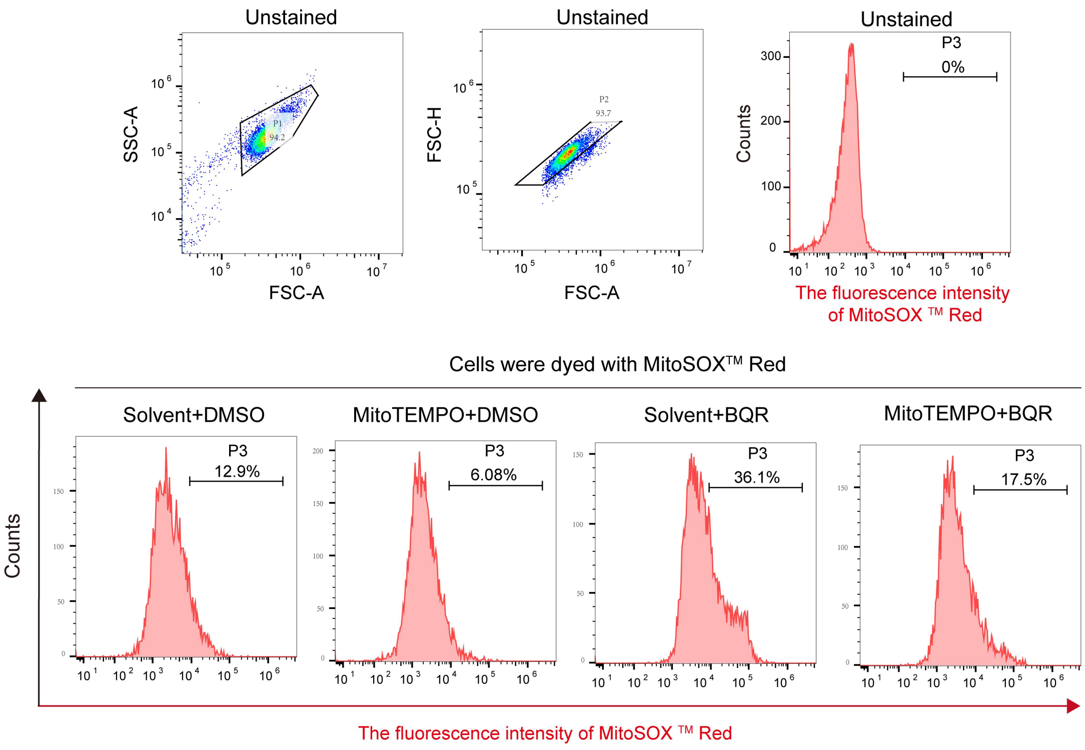

Figure S3. Schematic of the flow cytometric gating and analytical logic for mtROS measurement
Flow cytometric gating logic of mtROS measured by MitoSOX^{TM} Red staining. HeLa cells were pretreated with vehicle or the mtROS scavenger MitoTEMPO (10 µM) for 12 hours, followed by treatment with DMSO or 10 µM BQR for 24 hours.

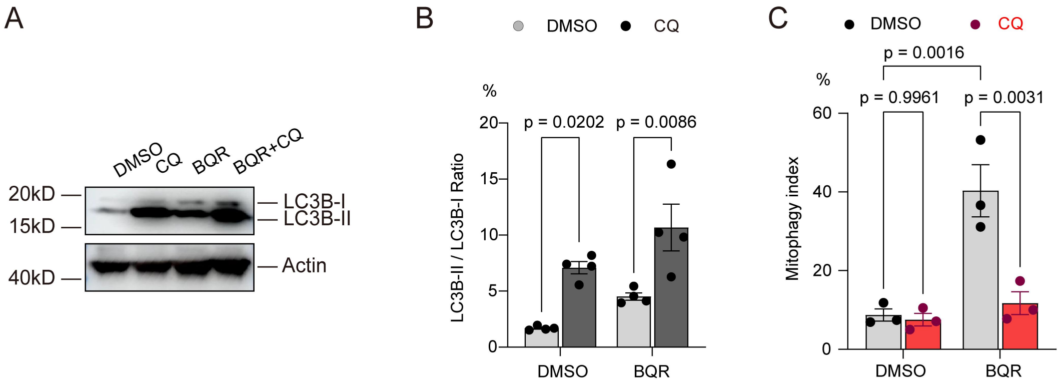

Figure S4. CQ treatment blocks both autophagy and mitophagy.
(A, B) Representative Western blot and quantification of autophagic markers. (A) Representative Western blot analysis of LC3B in HeLa cells treated with DMSO (vehicle), CQ (10 µM), BQR (10 µM), or the combination of BQR and CQ for 24 hours. β-Actin was used as a loading control. (B) Quantification of the LC3B-II/I ratio. $n = 4$ independent experiments.
(C) Flow cytometric quantification of mitophagy in HeLa cells expressing mt-Keima treated with DMSO (vehicle), CQ (10 µM), BQR (10 µM), or the combination of BQR and CQ for 24 hours. $n = 3$ independent experiments.
Statistical significance was determined by two-way ANOVA. p-values are indicated.

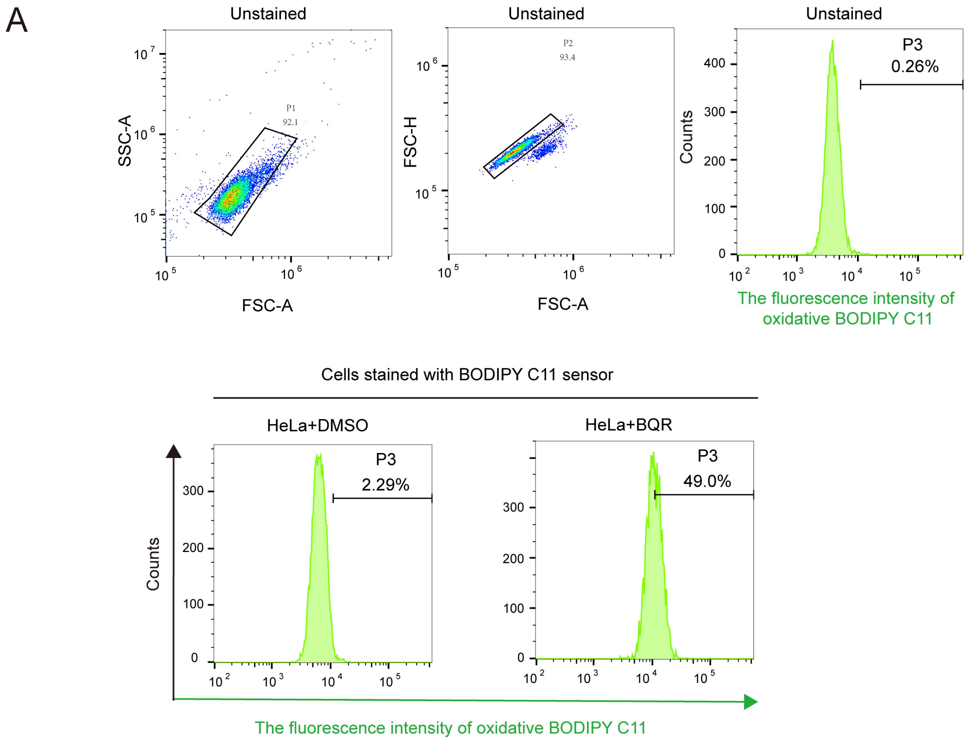

Figure S5. Schematic of the flow cytometric gating and analytical logic for lipid peroxidation measurement

Flow cytometric gating logic of lipid peroxidation measured by BODIPY™ C11 sensor. HeLa cells were treated with DMSO or 10 µM BQR for 24 hours.

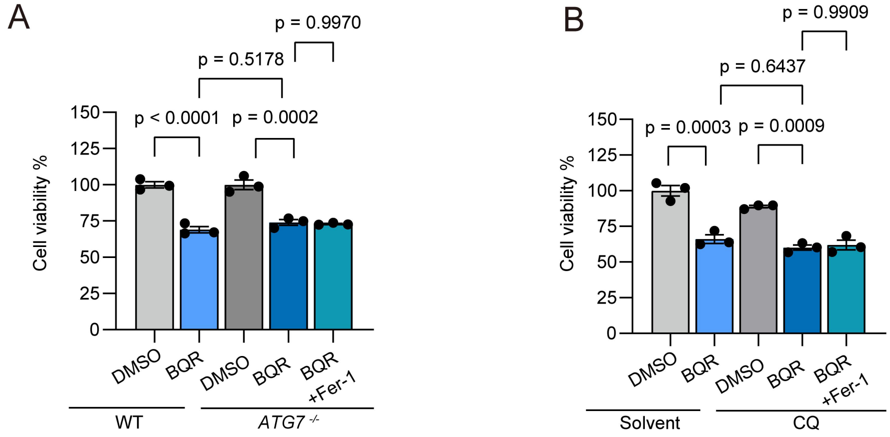

Figure S6. BQR shows minimal cytotoxicity with *ATG7* knockout or CQ treatment at 24 hours. **(A, B)** Cell viability analysis by CCK-8 assay at 24 hours, an earlier time point corresponding to the mtROS and lipid peroxidation assays. Experimental conditions mirror those in Figure 5A and 5B. n = 3 independent experiments.

Data are presented as mean ± SEM from at least three independent experiments. Statistical significance was determined by two-way ANOVA. p-values are indicated.

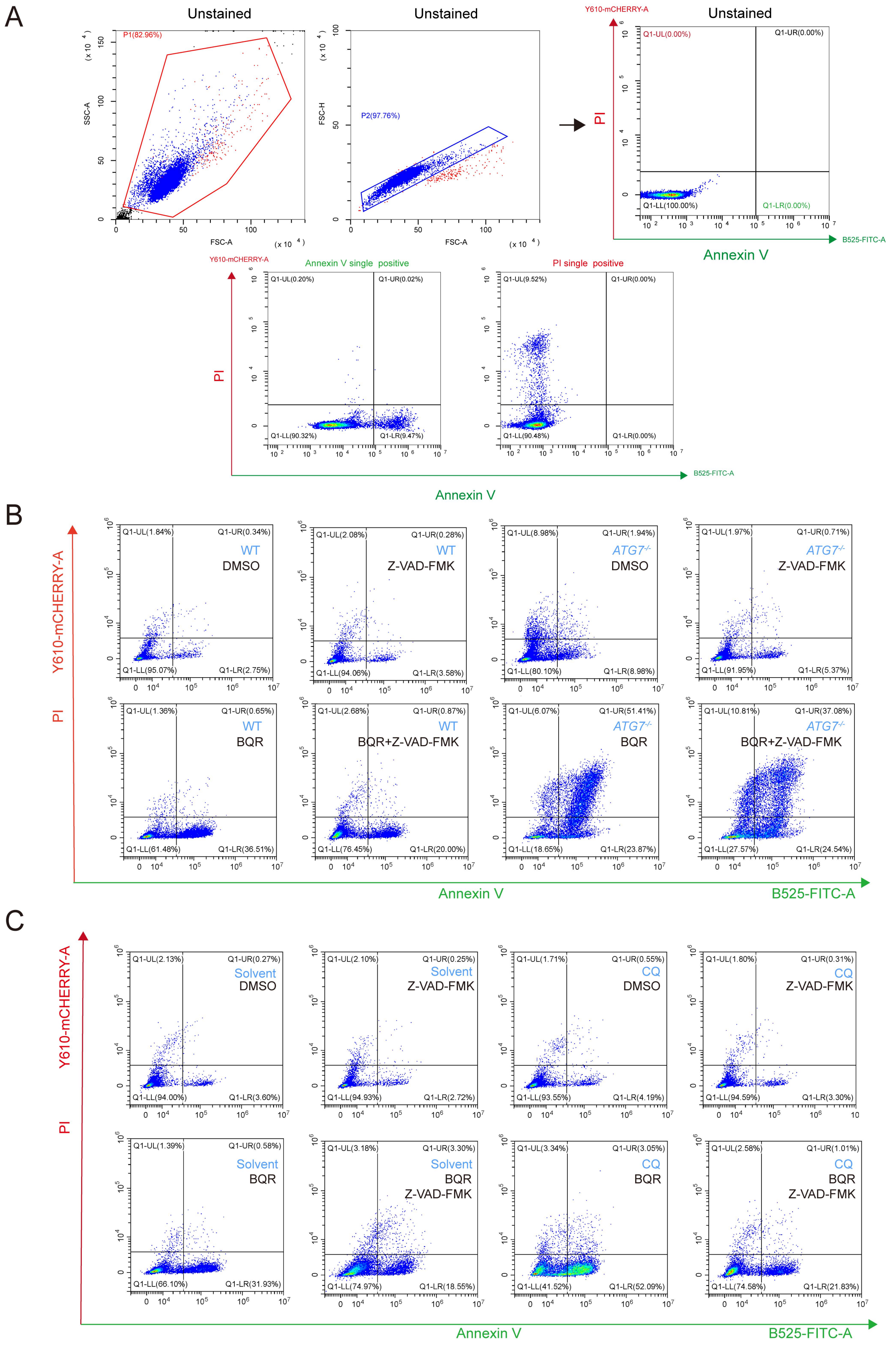
A
Unstained
P1(82.96%)
P2(97.76%)
FSC-A
SSC-A
FSC-H
Annexin V single positive
PI single positive
Y610-mCHERRY-A
B525-FITC-A
PI
Annexin V
B
WT
DMSO
Z-VAD-FMK
ATG7-/-
BQR
BQR+Z-VAD-FMK
C
Solvent
CQ
BQR
Z-VAD-FMK

Figure S7. Blockade of autophagic process enhances BQR-induced apoptosis.
(A) Schematic of Annexin V/PI staining logic for defining apoptotic cell populations. Representation of the logic used to define cell death stages based on Annexin V and PI fluorescence. Annexin V^-/PI^-: viable cells. Annexin V^+/PI^-: early apoptotic cells. Annexin V^+/PI^+: late apoptotic cells. Annexin V^-/PI^+: necrotic or damaged cells.
(B) WT and *ATG7*$^{-/-}$ HeLa cells were treated with 10 μM BQR for 48 hours, with or without the pan-caspase inhibitor Z-VAD-FMK (50 μM). Representative flow cytometry plots are shown.
(C) WT HeLa cells were treated with BQR and Z-VAD-FMK as in (B), with the addition of CQ (10 μM). Representative flow cytometry plots are shown.

Uncropped Western blots images

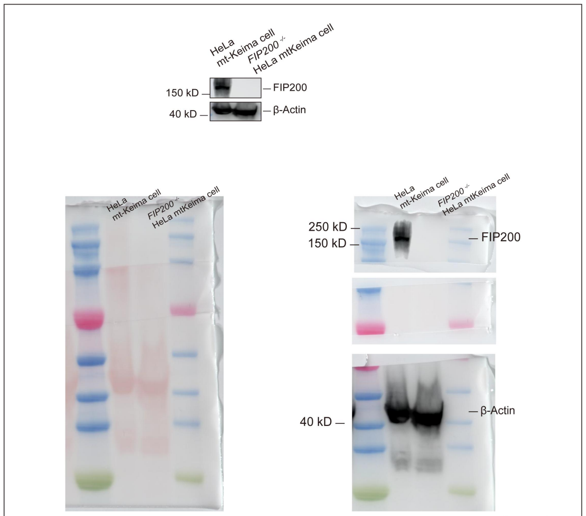

Figure 2A

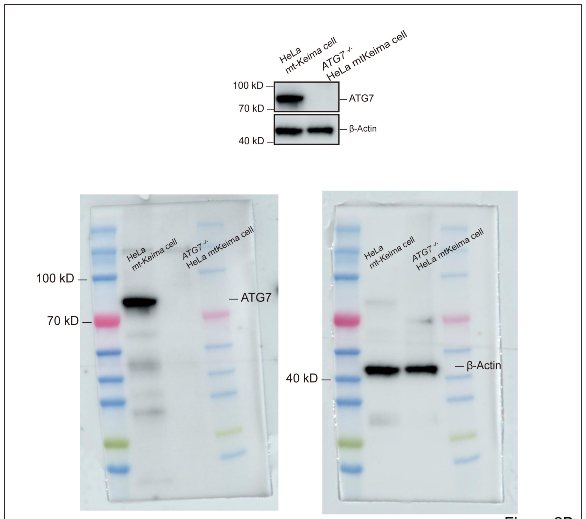

Figure 2B

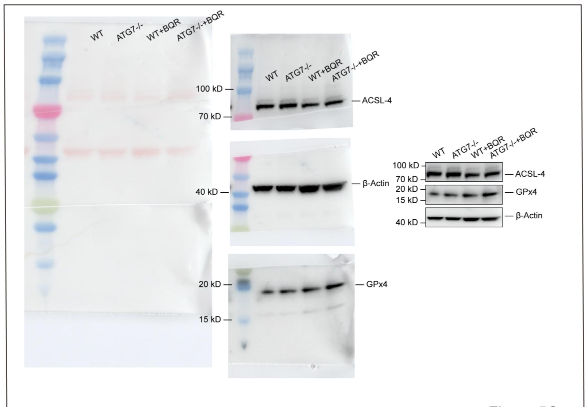

Figure 5C

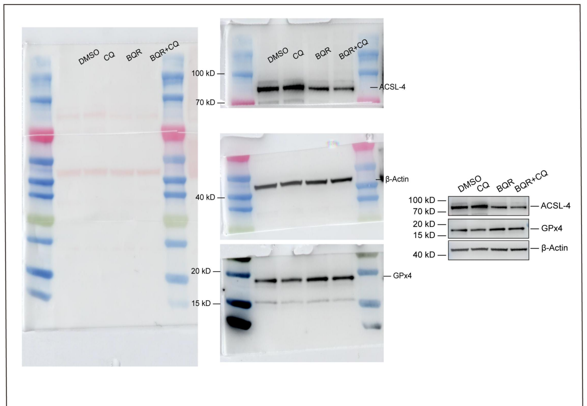

Figure 5D

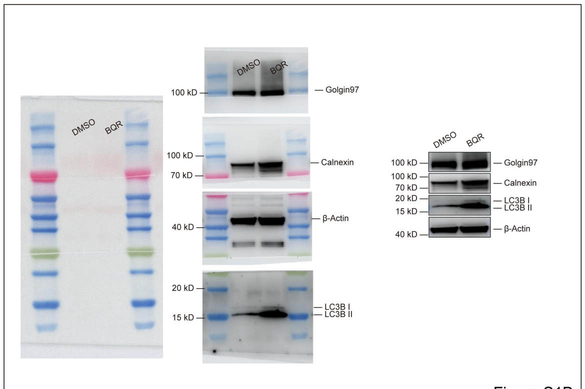

Figure S1B

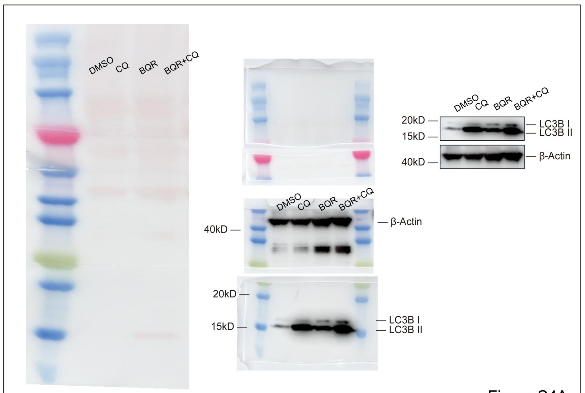

Figure S4A
